# Attributes of children and adolescents with avoidant/restrictive food intake disorder

**DOI:** 10.1186/s40337-019-0261-3

**Published:** 2019-09-12

**Authors:** Helene Keery, Sarah LeMay-Russell, Timothy L. Barnes, Sarah Eckhardt, Carol B. Peterson, Julie Lesser, Sasha Gorrell, Daniel Le Grange

**Affiliations:** 1 Center for the Treatment of Eating Disorders, Children’s Minnesota, Minneapolis, MN USA; 2 Children’s Minnesota Research Institute, Children’s Minnesota, Minneapolis, MN USA; 3 Department of Psychiatry, University of Minnesota, Minneapolis, MN USA; 4 The Emily Program, St. Paul, MN USA; 5 Department of Psychiatry, University of California, San Francisco, CA USA; 6 Department of Psychiatry and Behavioral Neuroscience, The University of Chicago, Chicago, IL USA

**Keywords:** Avoidant/restrictive food intake disorder, Anorexia nervosa, Atypical anorexia nervosa, Pediatric eating disorder

## Abstract

**Background:**

Avoidant/Restrictive Food Intake Disorder (ARFID) is a comparatively new DSM-5 diagnosis. In an effort to better understand this heterogeneous patient group, this study aimed to describe the physical and psychological attributes of children and adolescents with ARFID, and to compare them to patients with full-threshold or atypical anorexia nervosa (AN).

**Methods:**

Children and adolescents aged 7-to-19 years (*N* = 193) were examined upon presenting at a pediatric eating disorder center between July 2015 and December 2017. Data included diagnosis assessed via the semi-structured Eating Disorder Examination interview along with measures of anthropometrics, depression, anxiety, self-esteem, perfectionism and clinical impairment.

**Results:**

Compared to AN and atypical AN (*n* = 87), patients with ARFID (*n* = 106) were significantly younger (12.4 vs. 15.1 years, *p* < .0001), male (41% vs. 15%, *p* < .0002), and were more likely to be diagnosed with at least one co-morbid DSM-5 diagnosis (75% vs. 61%, *p* = .04). Patients with ARFID were less likely to be bradycardic (4.7% vs. 24.1%, *p* < .0001), amenorrheic (11.1 and 34.7%, *p* = .001), admitted to the hospital (14.2% vs. 27.6%, *p* = .02), and have a diagnosis of depression (18.9% vs. 48.3%, *p* < .0001). Patients with ARFID were significantly less likely to experience acute weight loss vs. chronic weight loss as compared with those with AN or atypical AN (*p* = .0001). On self-report measures, patients with ARFID reported significantly fewer symptoms of depression, anxiety, perfectionism, clinical impairment, concerns about weight and shape, and higher self-esteem than patients with AN or atypical AN (all *p*s < .0001). No differences were observed by race, anxiety disorder, orthostatic instability, suicidal ideation, and history of eating disorder treatment.

**Conclusions:**

Study results highlight the clinical significance of ARFID as a distinct DSM-5 diagnosis and the physical and psychological differences between ARFID and AN/atypical AN. The novel finding that ARFID patients are more likely than those diagnosed with AN to experience chronic, rather than acute, weight loss suggests important related treatment considerations.

## Plain English summary

This study describes physical and psychological attributes of children and adolescents with DSM-5 Avoidant/Restrictive Food Intake Disorder (ARFID) and compares them to patients with full-threshold or atypical anorexia nervosa (AN). Estimates of the percentage of patients with ARFID range from 5 to 41%. Comparisons to patients with AN have yielded inconsistent findings, but generally indicate that patients with ARFID are more likely to be younger, male, and have longer duration of illness. This is the largest study (*n* = 106) describing patients diagnosed with ARFID using formal DSM-5 criteria. By comparing patients with ARFID to those with AN/atypical AN, this study supports the separation of the diagnosis from other eating and feeding disorders. Findings also underscore the critical necessity of better understanding this unique patient population, which will ultimately improve early detection, and treatment efforts.

## Introduction

Avoidant/Restrictive Food Intake Disorder (ARFID) is a comparatively new eating disorder diagnosis in the Diagnostic and Statistical Manual of Mental Disorder, Fifth Edition (DSM-5) [1]. Historically, while patients with Food Avoidance Emotional Disorders were distinguishable from patients with anorexia nervosa (AN) thirty years ago [2], still relatively little is known about this heterogeneous group of patients and the extent to which ARFID may be similar or different from other types of restricting eating disorders [3]. ARFID was included in the DSM-5 to better capture the range of developmental feeding and eating problems as well as to more accurately diagnose patients who previously were diagnosed with an Eating Disorder Not Otherwise Specified or Feeding Disorder of Infancy or Early Childhood in earlier versions of the DSM [4, 5]. While patients with ARFID often present as underweight due to either long-term malnutrition or restriction of intake that causes a significant amount of weight loss, they do not experience the body image disturbance that is characteristic of patients with AN or atypical AN. Accumulating clinical data also suggest that ARFID may have distinct “subtypes.” However, these subtypes have not been formalized, and different studies have proposed varying categorizations, including selective eating, generalized anxiety, medical, food allergies, Autism Spectrum/Developmental Disorder, and food avoidant emotional disorder [1, 6–8], and more recently, the three categories of limited intake, limited variety, and aversive [9].

Recent research has provided information about the prevalence of ARFID as well as how children and adolescents with this diagnosis are similar to or different from those with AN. One study estimated the prevalence of ARFID in school children aged 8–13 years at 3.2% [10]. In outpatient adolescent medicine eating disorder clinics, estimates of patients diagnosed with ARFID range from 5 to 41% [3, 11–13]. Another study found that the prevalence of ARFID was 39% in an adolescent eating disorder day treatment program [14], and even higher (43%) in a sample of patients aged 6–12 years presenting with significant weight loss to an eating disorder clinic [15]. Comparisons to patients with AN generally indicate that ARFID patients are more likely to be younger, male, and have a longer duration of illness [3, 13, 15]. ARFID patients also have been found to be equally or more likely to have an anxiety disorder diagnosis and less likely to have a diagnosis of depression than those with AN [3, 14]. Finally, patients who fit criteria for ARFID tend to be underweight, although not to the same degree as patients with AN [3, 11, 14, 15].

Most of the previous studies comparing ARFID and AN/atypical AN have been retrospective chart reviews and used the proposed DSM-5 criteria for ARFID [3, 11–14]. A retrospective chart review published in 2018 used the formal DSM-5 criteria to identify 31 patients (8.4%) with an ARFID diagnosis but did not compare these patients with another group (i.e., AN) [16]. A recent overview of patients with ARFID in tertiary care was restricted by the younger age of its sample (i.e., under age 13) [17]. A description of patient characteristics based prospectively on the current criteria is warranted as this diagnosis gains more attention. Efforts to enhance our understanding of a still largely unknown diagnostic syndrome are of critical importance in improving early detection of ARFID, as well as in informing the development of tailored treatment interventions for this unique disorder. The aim of the present study was to compare ARFID with other restrictive eating disorders (AN and atypical AN) among patients presenting at a specialist community-based eating disorder service. This objective placed ARFID in context with broader eating disorder diagnoses, used both physical and psychological measures, and assessed patients at the time of presentation using DSM-5 criteria.

## Methods

This study examines baseline data from a longitudinal study designed to evaluate the effectiveness of evidence-based outpatient treatments for eating disorders. This study was approved by the Children’s Minnesota Institutional Review Board.

### Setting and participants

The Center for the Treatment of Eating Disorders (CTED) is a service within Children’s Minnesota that offers a multi-disciplinary approach to provide specialized care for eating disorders. The outpatient clinic treats patients aged 7 years and older, while the inpatient service treats those aged 7–21 years. Over 250 patients present for evaluation at CTED annually. Owing to a known special interest in treating ARFID, CTED receives increased referrals for this specific patient population from local medical providers (e.g., gastrointestinal specialists).

To be eligible for participation in the study, a patient had to: 1) be 7–19 years old; 2) present for an intake evaluation of eating disorder symptoms; 3) be diagnosed with a DSM-5 feeding or eating disorder; and 4) agree to participate in outpatient treatment. Study data were collected between July 2015 and December 2017. All eligible patients presenting for treatment were approached for participation in the study, and after obtaining informed consent (assent for patients < 18 years), research staff collected data from patients via interviews, pen-and-paper assessments, and electronic health records.

### Assessment and measures

Enrolled patients were evaluated by a psychiatrist, psychologist or clinical social worker, and trained assessors who administered measures at the time of the family’s first encounter with our service (inpatient or outpatient). Patients were admitted to the inpatient unit if they were determined to be medically unstable by the attending psychiatrist according to recommended guidelines of the American Academy of Pediatrics and the Society of Adolescent Medicine [18].

Patients completed age-appropriate self-report measures, and parents were given standardized assessments to complete about their child. The *intake questionnaire* included questions about eating disorder treatment history, current psychotropic medications, and medical history together with other medical conditions, including gastrointestinal and endocrine disorders. To determine eating disorder diagnosis, patients ages 12 years and older completed the EDE [19], which was also used to assess eating disorder symptom frequency severity as well as concerns about eating, weight, and shape. Patients younger than 12 years old were given a diagnosis agreed upon by the clinicians involved in the assessment after their diagnostic evaluations. Psychiatric assessment included *MINI-Kid* [20] to evaluate self-harm and suicidal ideation, the *Child Depression Inventory 2nd Edition* (CDI-2 [21]) to assess depressive symptoms, and the *Rosenberg Self-Esteem Scale* (RSE [22]) to assess self-esteem. The *Beck Anxiety Inventory* (BAI) [23] was used to assess anxiety, the *Clinical Impairment Assessment* [24] (CIA) to assess the psychosocial impact of the eating disorder, and the *Clinical Perfectionism Questionnaire* (CPQ [25]) to assess perfectionism outside of eating, weight or appearance. Parent reports of their child’s anxiety and depression were assessed using the internalizing scale of the *Child Behavior Check List* (CBCL) [26].

DSM-5 eating disorder and comorbid diagnoses were agreed upon by providers based on results from the EDE, semi-structured diagnostic assessment, MINI-Kid, and patient/parent report of symptoms. When patient and parent report differed, objective medical data regarding growth, weight loss, and medical stability were used to inform the diagnostic decision.

Medical assessment, including orthostatic vitals (supine and standing pulse rate and blood pressure), and weight were measured by a medical assistant or nurse. Bradycardia was defined as supine pulse rate of < 50 beats per minute, hypotension as < 90 mmHG, and orthostatic instability as difference in supine and standing heart rate of > 20 beats per minute or difference of > 10 mm HG in supine and standing blood pressure [17]. Amenorrhea was defined as the absence of menarche in girls age 15 years or older (primary) or the lack of menses for the past 3 months (secondary) [27].

AN cases were identified using a weight threshold of < 89% of expected body weight (%mBMI = BMI/50th percentile BMI for age and gender × 100) [28]. Patients who met all DSM-5 diagnostic criteria for AN and had a weight of > 90%mBMI were identified as atypical AN [29]. There was no upper level cut-off in BMI for an atypical AN diagnosis.

### Statistical analyses

All demographic, medical, and psychological measures were calculated as means and standard deviations for continuous variables and proportions for categorical variables. Initial analyses for demographic and weight presentation consisted of comparing measures across all three diagnoses. Analysis of variance (ANOVA) was applied for continuous variables (e.g., age, weight, and BMI) and χ2 or Fisher’s exact test was used, when appropriate, for categorical variables. Post hoc pairwise comparisons between individual eating disorder groups were also utilized to denote significance. As demonstrated by previous analyses by Sawyer and colleagues [30], data suggested that patients with AN and atypical AN differ by weight at presentation but do not differ significantly on other variables of interest. Further, although patients with AN and atypical AN differed significantly on weight presentation in our cohort, their scores on other medical and psychological measures did not differ significantly. As a result, and due to concerns about small sample sizes in stratified analyses, all further analyses compared the ARFID group to the AN and atypical AN patients combined (AN/atypical AN group; *n* = 87). Patients with ARFID were compared with those with AN and atypical AN by using independent *t*-tests for continuous variables (mean difference) and χ2 or Fisher’s exact tests for categorical variables (odds ratios). Between-group differences were assessed with a significance threshold of *p* < .05. All statistical analyses were performed in SAS 9.4 (Cary, NC).

## Results

Of the 404 patients who presented for evaluation at CTED during the study period, 338 (84%) met initial eligibility. After evaluation, 266 (66%) were verified as eligible, of which 243 patients were diagnosed with a DSM-5 eating disorder and enrolled in the present study. Of the 243 enrolled patients, 54 patients were diagnosed with AN, 36 with atypical AN, and 111 patients with ARFID (Table 1). Of those enrolled, certain exclusion criteria precluded their inclusion in analyses. Specifically, after accounting for individuals with binge/purge eating disorders who were not underweight (*n* = 17), individuals diagnosed with unspecified feeding and eating disorders (*n* = 24), and patients with missing data (*n* = 8), the final data set for analyses consisted of 193 patients. Within the 193 cases included in analyses, 52 (26.9%) met criteria for AN, 35 (18.1%) met criteria for atypical AN, and 106 (54.9%) met criteria for ARFID. Demographic characteristics are presented in Table 2. The mean age for patients with ARFID was 12.4 years compared to 15.1 years for AN and 15.2 years for atypical AN groups, respectively (*p* < .0001). Over 40% of patients with ARFID were male, compared to 15 and 11% of patients with AN/atypical AN (*p* = .0002). Most patients (≥ 80%) were White/Caucasian, with no significant differences across racial groups except for Hispanic ethnicity. When examining weight, patients with ARFID presented with a lower weight (37.6 kg, *SD* = 12.0 kg) on average compared to the AN/atypical AN group (44.4 kg, *SD* = 8.5 kg, and 56.7 kg, *SD* = 7.5 kg, respectively). In addition, the ARFID group presented with a significantly lower BMI, BMI z-score, and %mBMI compared to the AN/atypical AN group. While lower weight might be expected given the lower average age of those diagnosed with ARFID, these patients were the least likely to experience acute weight loss (vs. chronic weight loss) (14.9%), as compared with AN (40.0%) and atypical AN (50.0%) (*p* = .0001).

**Table 1:** Distribution of total eating disorder diagnoses at CTED, July 2015 – December 2017 (*n* = 243)

Diagnosis	*n*	%
Anorexia Nervosa (AN)	54	22.2
Bulimia Nervosa (BN)	9	3.7
Binge Eating Disorder (BED)	2	0.8
Avoidant/Restrictive Food Intake (ARFID)	111	45.7
Other specified feeding or eating disorder		
Atypical AN	36	14.8
AN: limited body image/ behaviors	–	–
BN: low frequency duration	1	0.4
BED: low frequency duration	1	0.4
Purging Disorder	4	1.6
Unspecified Feeding or Eating Disorder	24	9.9

**Table 2:** Demographic and weight presentation of study participants with AN, Atypical AN, and ARFID (*n* = 193)

	AN *n* = 52	Atypical AN *n* = 35	ARFID *n* = 106	*p* ^*a*^
	*M* (*SD*) or *n* (%)	*M* (*SD*) or *n* (%)	*M* (*SD*) or *n* (%)	
Age	15.06 (1.96)^*^	15.23 (1.88)^*^	12.39 (2.65)^**^	<.0001
Gender				
Male	8 (15.38)^*^	4 (11.43)^*^	43 (40.57)^**^	.0002
Female	44 (84.62)	31 (88.57)	63 (59.43)	
Race/Ethnicity				
White/Caucasian	45 (86.54)	28 (80.00)	87 (82.08)	.69
Black/African-American	0 (0)	1 (2.86)	2 (1.89)	.75^b^
Hispanic/Latino	0 (0)^**^	6 (17.14)^*^	9 (8.49)^*^	.005^b^
Asian	4 (7.69)	0 (0)	4 (3.77)	.25^b^
Weight Presentation				
Kilograms	44.41 (8.50)^*^	56.67 (7.50)^**^	37.61 (11.97)^***^	<.0001
BMI	16.63 (1.74) ^*^	20.66 (2.11)^**^	16.09 (2.67)^***^	<.0001
BMI z-score	−1.59 (1.00)^*^	0.21 (0.62)^**^	−1.49 (1.45)^*^	<.0001
%mBMI	83.11 (7.75)^*^	103.33 (11.54)^**^	86.40 (14.87)^*^	<.0001
Acute Weight Loss (vs. Chronic)^c^	16 (40.0%)^*^	12 (50.0%)^*^	15 (14.9%)^**^	.0001

^a^Analyzed using Pearson’s Chi-square test for categorical variables or ANOVA for continuous variables, unless noted differently. ^b^ Due to small cell count, analyzed using Fischer’s Exact test. ^c^ Data missing for 28 patients (for AN: *n* = 12, for Atypical AN: *n* = 11, and for ARFID: *n* = 5). Race/Ethnicity excludes patients who declined to respond or selected “other” (*n* = 7). ^*/**/***^ Denotes significance between eating disorder groups using pairwise comparisons (*p* < .05)

When comparing patients with ARFID to the AN/atypical AN group, significant differences between vital signs and medical instability were observed (Table 3). Pulse rate (78.8 vs. 63.4, *p* < .0001) and systolic blood pressure (113.1 vs. 109.7, *p =* .02) were all significantly higher for the ARFID than for the AN/atypical AN group. The ARFID group was significantly less likely to be bradycardic (OR = 0.16, *p* < .0001), amenorrheic (OR = 0.24, *p* = .001), and admitted to the hospital (OR = 0.43, *p* < .02) compared to the AN/atypical AN group. ARFID and AN/atypical AN groups did not differ on measures of hypotension, orthostatic instability, or history of prior eating disorder treatment (all *p*s > .05).

**Table 3:** Medical presentation of study participants with AN/Atypical AN, and ARFID (*n* = 193)

	AN or Atypical AN (*n* = 87)	ARFID (*n* = 106)	OR or Mean Difference	*P* ^*a*^
	*M* (*SD*) or *n* (%)	*M* (*SD*) or *n* (%)		
Vital Signs				
Pulse rate	63.44 (16.62)	78.80 (15.91)	−15.35	< .0001
Systolic blood pressure	109.7 (12.09)	113.1 (9.42)	−3.35	.04
Diastolic blood pressure	61.57 (6.71)	63.43 (7.70)	−1.86	.07
Medical Instability				
Bradycardia, < 50 beats per minute	21 (24.14)	5 (4.72)	0.16	< .0001^b^
Hypotension, systolic pressure < 90 mmHg	4 (4.60)	2 (1.89)	0.40	.41^b^
Orthostatic Instability, > 20 beats per minute, > 10 mmHg	48 (55.17)	57 (53.77)	0.95	.85
Admitted/In hospital at presentation	24 (27.59)	15 (14.15)	0.43	.02
Amenorrhea^c^	26 (34.67)	7 (11.11)	0.24	.001
Prior Eating Disorder Treatment	33 (37.93)	40 (37.74)	0.99	.98
Psychotropic medication	34 (39.08)	38 (35.85)	0.87	.64
Other medical condition present potentially impacting weight^d^	7 (8.05)	13 (12.26)	1.60	.34

^a^Analyzed using Pearson’s Chi-square test for categorical variables or independent t-tests for continuous variables, unless noted differently. ^b^ Due to small cell count, analyzed using Fischer’s Exact test. ^c^ Excludes males (for AN/atypical AN, 12 excluded; for ARFID, 43 excluded). ^d^ Conditions considered included gastrointestinal or endocrine disorders including, but not limited to delayed gastric emptying, gastritis, esophagitis, GERD, Grave’s disease, Type 1 Diabetes, chronic constipation, food allergies, acid reflux, celiac disease, irritable bowel syndrome, hypothyroidism, Hashimoto’s disease, and gastroparesis

Using χ2 analyses, patients with ARFID were less likely than the AN/atypical AN group to be diagnosed with depression (OR = 0.25, *p* < .0001), but more likely to have an existing psychiatric comorbidity (OR = 1.88, *p* = .04) (Table 4). Furthermore, nearly 24% of patients with ARFID had a diagnosis of attention-deficit/hyperactivity disorder (ADHD) compared to only 1.15% in the AN/atypical AN group (*p* < .0001). Compared to the AN/atypical AN group, patients with ARFID scored significantly lower on the Global EDE (0.34 vs. 2.56, *p* < .0001) and corresponding subscales. In addition, patients with ARFID scored significantly lower on the self -report CDI-2 (53.2 vs. 62.0, *p* < .0001), the CPQ (19.4 vs. 25.8, *p* < .0001), the CIA (10.2 vs. 22.4, *p* < .0001), and BAI Total Score (10.2 vs. 16.1, *p* = .002). Patients with ARFID also scored significantly higher on the Rosenberg Self-Esteem (21.9 vs. 15.2, *p* < .0001) compared to the AN/atypical AN group. There were no statistical significant differences on the parental report of CBCL Internalizing T-Score (61.8 vs. 63.6, *p* = .34) or self-report on self-harm/suicidal ideation (9.4 vs. 13.6, *p* = .47).

**Table 4:** Psychiatric measures of participants with AN/Atypical AN, and ARFID (*n* = 193)

	AN or Atypical AN (*n* = 87)	ARFID (*n* = 106)	OR or Mean Difference	*p* ^*a*^
	*M* (SD) or *n* (%)	*M* (*SD*) or *n* (%)		
Diagnoses				
Psychiatric comorbidity (any DSM-V diagnosis)	53 (60.92)	79 (74.53)	1.88	.04
ADHD	1 (1.15)	25 (23.58)	26.54	< .0001^b^
Autism	1 (1.15)	6 (5.66)	5.16	.13^b^
Depressive Disorder^c^	42 (48.28)	20 (18.87)	0.25	< .0001
Anxiety Disorder^d^	41 (47.13)	61 (57.55)	1.52	.15
Eating Disorder Examination^e^				
Global score	2.56 (1.58)	0.30 (0.34)	2.25	< .0001
Restraint	2.83 (1.77)	0.29 (0.59)	2.55	< .0001
Eating concerns	1.66 (1.52)	0.32 (0.55)	1.33	< .0001
Shape concerns	2.61 (1.83)	0.33 (0.53)	2.28	< .0001
Weight concerns	3.11 (1.95)	0.27 (0.43)	2.85	< .0001
Parent Report				
CBCL Internalizing T-Score^f^	63.56 (10.22)	61.84 (11.53)	1.73	.34
Self-Report Measures				
vSelf-harm/ Suicidal ideation (MiniKid)^f^	10 (15.63)	8 (10.00)	0.60	.31
Children’s Depression Inventory (Total T Score)^f^	61.97 (14.59)	53.18 (10.56)	7.87	< .0001
Rosenberg Self-Esteem^f^	15.19 (7.85)	21.85 (6.06)	−6.66	< .0001
Beck Anxiety Inventory Total Score^e^	16.07 (11.50)	10.17 (9.24)	5.90	.001
Clinical Impairment Assessment^e^	22.39 (13.03)	10.15 (8.98)	11.24	< .0001
Clinical Perfectionism Questionnaire^e^	25.75 (6.85)	19.38 (4.84)	6.37	< .0001

^a^Analyzed using Pearson’s Chi-square test or for categorical variables or independent t-tests for continuous variables. ^b^ Due to small cell count, analyzed using Fischer’s Exact test. ^c^ Patient is considered to have diagnosis of depression if they were diagnosed with Dysthymia, Major Depressive Disorder, Other Specified Depressive Disorder, and/or Unspecified Depression. ^d^ Patient is considered to have an anxiety diagnosis consists if they were diagnosed with OCD, PTSD, Social Anxiety Disorder, Agoraphobia, Generalized Anxiety Disorder, Panic Disorder, Separation Anxiety Disorder, Specific Phobia, Other Specified Anxiety Disorder, and/or Unspecified Anxiety. ^e^ Restricted to patients age ≥ 12 (for AN: *n* = 81, for ARFID: *n* = 63) and existing values for EDE subscales, BAI, CIA, and BAI (for AN: *n* = 75, for ARFID: *n* = 53). ^f^ Removed missing values for internalizing *t*-score, self-harm, total *t*-score, and RSE (for AN: 23 excluded, for ARFID: 26 excluded)

## Discussion

The aim of this study was to provide a comprehensive comparison of the unique physical and psychological presentation of patients diagnosed with ARFID according to DSM-5 guidelines when compared to patients with AN and atypical AN. First, this study found that while smaller stature may be expected given the younger age of this subgroup, ARFID patients were significantly lower in weight than both AN/atypical AN patients. Additionally, patients with ARFID were equally orthostatic as the AN/atypical AN group, but were less likely to be amenorrheic, admitted to the hospital, or on psychiatric medication. While patients with AN were more likely to be older and female, which likely affected comparative rates of amenorrhea among those with ARFID, overall, the physical differences identified in the current study highlight the importance of thorough medical assessments to determine the impact of low presenting weight. In one recent study, latent class analysis was used to silo eating disorder symptoms into groups that reflected the DSM-5 diagnostic categories of AN and ARFID [31]. For the most part, the findings of Pinhas and colleagues [31] align with our results, regarding between-group differences in age, prior hospitalization, eating disorder symptomology, and rates of psychiatric comorbidity. However, in Pinhas et al. [31], AN patients were more likely to have unstable vital signs whereas in our sample, vital signs were comparable with the exception of bradycardia. Patients with ARFID in the current sample were also less likely to experience acute weight loss, suggesting that future study may benefit from examining rates of weight loss and related consequences for vital sign stability among children and adolescents with ARFID.

Given that the ARFID classification was more recently established in the DSM-5 [1], relatively little research has been published on this population to date. Previous studies describing this population have used retrospective chart reviews and/or assigned patients an ARFID diagnosis based on the proposed DSM-5 criteria [3, 11–15], while our data was prospectively collected and was based on a DSM-5 ARFID diagnosis via a clinician interaction with each patient. Other recent study using the latest diagnostic criteria has been limited by either a restricted age range (i.e., only those aged 13 and younger) [17] or had a substantially smaller sample (*n* = 31) and did not include a comparison group [16]. With an adequately powered sample across a broad age range (i.e., 7–19), our study shows consistency in weight status with previous literature that has characterized patients diagnosed either retrospectively or prospectively with ARFID. Of note, in the current study, patients with ARFID had a lower %mBMI than those diagnosed with AN, which lies in contrast to evidence from prior work indicating patients who fit criteria for ARFID tend to be less underweight than patients with AN [3, 11, 14, 15]. Our findings may be partially explained by the fact that those diagnosed with ARFID in the current sample were also on average, younger in age, and therefore expected to present smaller in stature. However, %mBMI is calculated based upon metrics correspondent to age and height, suggesting that our findings would benefit from replication in future investigation. Patients with ARFID in our sample were also more likely than the AN/atypical AN group to be younger and male [3, 12]. Diagnosis with a comorbid DSM-5 disorder was a common feature between our findings and those of other studies as well [3, 16]. With regard to our inclusion of atypical AN, we saw similarity to a previous study; patients in our study with AN and atypical AN were observed to have similar levels of psychopathology despite the significant differences in their %mBMI [30]. Notably, those with ARFID were significantly more likely to be diagnosed with ADHD than either AN or atypical AN. While this difference is perhaps reflective of elevated population rates of ADHD among males, future research might investigate this specific comorbidty as a clinical feature of ARFID, with particular attention paid to the impact of stimulant medication use on appetite and clinical presentation. Further, given the significantly higher rates of ADHD evidenced among those diagnosed with ARFID in the current study, and the known comorbidity between ADHD and Autism Spectrum/Developmental Disorder, future work should more specifically investigate this potential shared diagnostic risk.

There are several limitations to this study. First, the sample is primarily white, which limits generalizability. Also, we combined AN and atypical AN because of power concerns; however, as demonstrated by Sawyer et al. [30] and our own analyses, no significant group differences were observed on medical and psychological variables. Further, due to the small sample size of children 12 and younger, we were not able to compare the children aged 12 and under on a measure of anxiety. Finally, lack of information on duration of illness did not allow for between-group comparisons. While the current study indicates that those with ARIFD were more likely to experience chronic vs. acute weight loss, it is noteworthy that assessment of duration of illness in ARFID patients can be difficult given that on average, patients meeting criteria for this disorder have extended duration of illness compared to patients diagnosed with AN, atypical AN and bulimia nervosa [3, 13, 15]. Further, assessment measures specifc to ARFID have been slow to emerge; while this study used a rigorous method with which to determine ARFID diagnosis, future study should rely upon empirically validated measures, specifically oriented toward a determination of this diagnosis.

The current study uniquely identified differences across disorders in acute vs. chronic weight loss. Specifically, those with ARFID were less likely to experience acute vs. chronic weight loss as compared with AN and atypical AN counterparts. Restrictive eating disorders and weight loss have a significant negative impact on both the physical and psychological functioning of children and adolescents [27]. Research suggests that in patients with AN, weight loss and malnutrition are associated with a number of significant life-threatening medical complications including hypotension, bradycardia, and hypothermia [32]. Weight loss in patients with AN has also been associated with atrophy of the brain and decreased cognitive functioning [32]. Similarly, Sawyer et al. demonstrated that adolescents diagnosed with atypical AN have similar medical and psychological impairments as patients with full-threshold AN [30]. However, there are not yet any published studies investigating the physical and psychological effects of weight loss and malnutrition in patients with ARFID; this would be an important area for future study following the descriptive work in the evidence base to date. Learning that patients with ARFID were significantly *less* likely to experience acute weight loss vs. chronic weight loss as compared with those with AN or atypical AN, may be critical in informing future screening efforts. For example, it is possible that a sizeable portion of ARFID patients might be missed if we focus only on those who present at low weight, or those who appear to have lost weight precipitously. Evidence that patients who meet criteria for ARFID are significantly younger than youth with AN and atypical AN, along with potential health consequences associated with sustained low weight, underscores a critical line of future inquiry in this unique patient population.

This study provides additional data to support the validity of separating ARFID from other eating and feeding disorders including AN and atypical AN. Given the relative newness of the diagnosis, ARFID patients may not be detected as early or often as those with more traditionally described AN/atypical AN, leading to reduced access to care. Early detection of ARFID may lead to faster mobilization of treatment efforts and potentially better illness prognoses. As such, our findings based on formal DSM-5 criteria in a sizable sample of individuals with ARFID provide an important foundation for future efforts to better define this clinical syndrome and to ultimately tailor precision interventions for this population.

## Conclusions

The current study was uniquely able to examine clinical features of a large treatment-seeking sample of children and adolescents diagnosed with ARFID. Findings from this study highlight the clinical significance of ARFID as a distinct DSM-5 diagnosis and describe the physiological and psychological presentation of this disorder. The novel finding that ARFID patients are more likely than those diagnosed with AN to experience chronic, rather than acute, weight loss should be further investigated, along with associated health consequences. Given the medical and psychiatric heterogeneity of this population, further research is also needed to examine potential differences between ARFID subgroups, as well as to development reliable and valid assessment measures for ARFID that can further improve understanding of this clinical syndrome. The current study provides an important foundation in understanding medical and psychological features of children and adolescents presenting for treatment with ARFID.

## Data Availability

The datasets used and analysed during the current study are available from the corresponding author on reasonable request.

## Abbreviations

ADHD: Attention-deficit/hyperactivity disorder
AN: Anorexia nervosa
ANOVA: Analysis of variance
ARFID: Avoidant restrictive food intake disorder
BAI: Beck Anxiety Inventory
BMI: Body mass index
CBCL: Child Behavior Check List
CDI-2: Child Depression Inventory 2nd Edition
CIA: Clinical Impairment Assessment
CPQ: Clinical Perfectionism Questionnaire
CTED: Center for the Treatment of Eating Disorders
DSM-5: Diagnostic and Statistical Manual of Mental Disorder, Fifth Edition
EDE: Eating Disorder Examination
FAED: Food avoidant emotional disorder
MINI-Kid: Mini International Neuropsychiatric Interview for Children and Adolescents
RSE: Rosenberg Self-Esteem Scale
